# CovHos, a New Score to Predict the Need of Hospitalization for Coronavirus Disease 2019 (COVID-19) Patients at the Emergency Department

**DOI:** 10.7759/cureus.18717

**Published:** 2021-10-12

**Authors:** Veronica Salvatore, Alice Gianstefani, Gabriele Farina, Ilaria Carletti, Nicoletta Carpentieri, Anna Laura Tinuper, Francesca Trabalza, Alice Grignaschi, Fabrizio Giostra

**Affiliations:** 1 Emergency Department, Medicina d'Urgenza e Pronto Soccorso, IRCCS Azienda Ospedaliero-Universitaria di Bologna, Bologna, ITA

**Keywords:** covid-19, coronavirus, emergency department, score, discharge

## Abstract

Introduction and aim: As first receivers of suspected coronavirus disease 2019 (COVID-19) patients, clinicians of the Emergency Department (ED) have to rapidly perform the first clinical assessment evaluating the intensity of care needed. So far, clear management guidelines still lack. We identified variables associated with hospitalization in order to give a quick tool to assist clinicians in stratifying cases based on the severity at their arrival at the ED and in predicting the need for hospital care.

Methods: This is a monocentric observational prospective study enrolling COVID-19 patients. A score for hospitalization prediction (CovHos Score) was created using variables associated with hospitalization at multivariate analysis and then validated on an internal subsequent cohort.

Results: A total of 667 patients were included; 465 (69.7%) were hospitalized and 108 (16.2%) died at 30-days follow-up. In a multivariate analysis, male sex, age>65, alveolar-to-arterial oxygen gradient percentage increase compared to that expected for age, neutrophils/lymphocytes ratio and C-reactive protein levels were significantly associated with a higher rate of hospital admission. A CovHos score cut-off of 12 points predicted hospitalization with 85% sensitivity and 82.4 % specificity (area under a receiver operating characteristic curve [AUROC] = 0.909, 95% CI 0.884 - 0.935). Similar results were obtained in the validation court. A cut-off of 22 has 79% sensitivity and 77% specificity in predicting mortality (AUROC = 0.824; 95% CI 0.782-0.866); sensitivity and specificity were respectively 71.4% and 71.3% in the validation group.

Conclusions: Although medical judgment still remains crucial, the CovHos score is an effective tool to assist emergency clinicians in predicting the need for hospitalization or to optimize allocation in a shortage of hospital resources.

## Introduction

In December 2019, a new disease called ‘Coronavirus disease 2019 (COVID-19) appeared, generated by an unknown enveloped RNA beta-coronavirus named severe acute respiratory syndrome coronavirus 2 (SARS-CoV-2) [1-3]. SARS-CoV-2 had a big impact, with an incidence of more than 34.8 million cases, causing more than 1 million deaths in the period going from the beginning of the epidemic to October 6th, 2020 [4] with a worldwide case fatality rate of about 12% [5]. The symptoms of COVID-19 in its clinical course are primarily respiratory, including cough, fever, and dyspnea; and in the most severe cases, it rapidly progresses to ARDS (Acute Respiratory Distress Syndrome) [6]. Arterial blood gas analysis (ABG) frequently highlights hypoxemic respiratory failure associated with respiratory alkalosis. Most patients typically reveal a discrepancy between clinical and ABG findings, presenting to the Emergency Department (ED) with extremely low partial pressure of arterial oxygen (PaO_2_) values not associated with dyspnea [7]. Moreover, a reduced partial pressure of arterial carbon dioxide (PaCO_2_) is often present, due to an increase in respiratory rate (RR) needed to maintain an adequate PaO_2_. Only a small proportion of patients will develop severe respiratory failure (SRF) but many of these require mechanical ventilation [8].

In Italy, from the beginning of the pandemic to October 5th, 2020, the number of people affected by COVID-19 was 327,586, with 36,002 deaths (0.11%) [9]. Italian hospitals were faced with a rising number of cases, with limited healthcare resources due to the sudden and ever-increasing pandemic. As first receivers of suspected COVID-19 patients, EDs have to rapidly perform the first clinical assessment and the identification of the most critical patients or those at worsening risk, evaluating the intensity of care needed. So far, ED physicians still lack clear management guidelines. According to literature, 14% of patients have severe disease and 5% a critical disease requiring intensive care while the majority of patients (81%) present with mild disease. Although emergency doctors are well trained to recognize and treat severe and critical COVID-19 patients, they are more likely to manage mild cases. Deciding, in an ED setting, whether patients with mild illness are suitable for home management or require in-hospital observation due to an increased worsening risk is challenging.

The aim of our study is to give a quick, reliable, and effective tool to assist clinicians in stratifying cases based on the severity at their arrival at the ED and in predicting the need for hospitalization, notably for unselected patients as in an emergency setting. We investigated COVID-19 features, as reported at the time of patient admission, in order to point out those variables associated with hospitalization. CovHos Score (COVID-19 score for hospitalization prediction) was thus created. CovHos score was then validated on a subsequent cohort.

To evaluate the severity of the respiratory failure and pulmonary impairment in patients affected by COVID-19, we used, among standard parameters, the Alveolar-to-arterial Oxygen Gradient (AaDO_2_). The AaDO_2_ measures the difference between the alveolar (pAO_2_) and arterial oxygen (PaO_2_) concentration, helping to determine the source of hypoxemia. In patients affected by pneumonia, where the physical barrier within the alveoli reduces the oxygen diffusion through the capillaries, AaDO_2_ is inappropriately high [10].

## Materials and methods

Study design and participants

From March 13th to April 4th, 2020, we conducted a monocentric observational prospective study enrolling consecutively all adult patients (≥ 18 years old), who came to the ED of Sant’Orsola-Malpighi Hospital (Bologna, Italy) showing suspicious signs and symptoms of COVID-19, like fever, cough, pharyngodynia, fatigue, anosmia, ageusia, conjunctivitis, headache, myalgias, diarrhoea, and syncope. All suspected cases of COVID-19 were admitted to a COVID area of our ED, where demographic, case history, and clinical data were collected, laboratory tests (ABG, general blood tests), and radiological exams (lung ultrasound, chest X-ray, and/or high resolution computed tomography [HRCT]) were performed. All patients underwent a nasal and pharyngeal swab for SARS-CoV-2. Out of all suspected patients admitted to ED, only those diagnosed with COVID-19 have been included in our analysis, both directly discharged and hospitalized patients. COVID-19 diagnoses have been established on a positive real-time reverse-transcription polymerase-chain-reaction (RT-PCR) assay for nasal and pharyngeal swab specimens and/or on typical imaging acquired by lung ultrasound, X-ray, or HRCT. Patients discharged or admitted to the hospital with an alternative diagnosis were excluded from the analysis. The study was approved by our local Ethics Committee (approval number: 551/2020/Oss/AOUBo). Verbal informed consent was obtained from all individual participants included in the study.

Epidemiological, demographic, clinical, laboratory, treatment, and outcome data were extracted from electronic medical records taken since the patient's arrival at the ED. Concerning the survival analysis, the 30-days all-cause mortality since the ED admission was considered. All patients have been contacted by telephone after 30 days in order to highlight a potential hospital re-admission other than Sant’Orsola-Malpighi Hospital.

Among the possible predictive variables for hospitalization, the following patient variables at hospital admission were included: demographic variables, medical history, clinical signs and symptoms, arterial blood gas, and laboratory findings. Besides, in order to evaluate the severity of the respiratory failure, we calculated AaDO_2_ and AaDO_2_ percentage increase compared to the expected value for a given age. AaDO_2_ was calculated using the formula:

$AaDO2 = [(FiO2)(Atmospheric\: pressure - H2O\: pressure) - (PaCO2/0.8)] - PaO2$

Normal gradient estimated = $(age/4) + 4$

Statistical analysis and score creation

General characteristics were reported as mean ± standard deviation (SD) and frequencies (percentages) for continuous and categorical variables, respectively. Comparisons between groups were performed using the Chi-square test and the Student’s t-test or Mann-Whitney test for independent groups, for categorical and continuous variables, respectively, where appropriate.

Logistic binary regression analysis was run, taking hospitalization as the outcome. Statistically significant variables at univariate analysis were included in multivariate analysis. The strength of the association between variables and hospitalization was reported as odds ratios (ORs) with their 95% confidence intervals (95% CI). We built up a scoring system based on such findings to properly select COVID-19 patients requiring in-hospital treatment. CovHos score was constructed based on the coefficients from the logistic model, as the sum of each variable multiplied by its own OR. Receiver Operating Characteristic (ROC) analysis was used to determine the accuracy of the CovHos score in predicting hospitalization and to find the corresponding cut-off value. A ROC analysis was also performed to assess CovHos score accuracy in predicting mortality. P values <0.05 were considered significant for all the tests. SPSS statistics version 25 (IBM Corp., Armonk, NY) was used for statistical analyses. 

Score validation

In order to validate the CovHos score, we tested it on a cohort of patients consecutively admitted to our ED from April 4th to April 30th that fulfilled the same inclusion and exclusion criteria. Exact Fisher’s test was used to assess the statistical significance of the CovHos score in the prediction of hospitalization and mortality. Specificity and sensitivity were calculated on 2x2 tables.

## Results

Out of 1,366 patients admitted to the COVID area of our ED, 667 were included in the analysis. A total of 465 (69.7%) were hospitalized and 108 (16.2%) died at 30-days follow-up. Out of 202 discharged patients, 27 came back to the ED within 30 days; 15 of them were discharged and 12 hospitalized (6%).

Features of the enrolled population

The mean ± SD age of our cohort was 61.9 ± 18.9 years and 355 (53.2%) were males. The most common comorbidities were hypertension (37.3%), diabetes (10.8%), and chronic obstructive pulmonary disease (9.9%). In 423 patients (63.4%), symptoms onset went back to less than six days before arriving at the ED and the most frequently observed symptoms were fever (81.7%), cough (55.6%), dyspnoea (37.3%), and fatigue (16.8%). Concerning the respiratory parameters, we have found that RR was 19.8 ± 5.4, SpO_2_ was 95.6 ± 4.4 and PaO_2_/FiO_2_ (P/F) was 347 ± 99.1. 

Hospitalized and non-survivors were significantly older (68.5 ± 16.7; 81.2 ± 10.6) than discharged patients and survivors (46.7 ± 14.4; 58.1 ± 17.9) and had a higher prevalence of hypertension (48% and 65.7%, respectively). At the time of ED admission, they also complained of a higher rate of dyspnoea (40.6% vs 29.7%; 57.4% vs 33.5% respectively), showing a higher RR and a lower SpO_2_. The full demographic and clinical specifications are included in Table 1.

**Table 1:** Demographic and baseline clinical characteristics Data are mean ± SD or n (%). COPD: chronic obstructive pulmonary disease. SpO_2_: peripheral oxygen saturation. ED: emergency department. MBP: mean arterial blood pressure. HR: heart rate. RR: respiratory rate. BT: body temperature. NS: not significant

Characteristics	All patients (n=667)	Discharged (n=202)	Hospitalized (n=465)	p-value	Survivors (n=559)	Non-survivors (n=108)	p-value	
Age, years	61.9 ±18.9	46.7 ± 14.4	68.5 ± 16.7	< .001	58.1 ± 17.9	81.2 ± 10.6	< .001	
Sex								
Male	355 (53.2)	86 (24.2)	269 (75.8)	< .001	291 (82)	64 (8)	< .001	
Female	312 (46.8)	116 (37.2)	196 (62.8)	< .001	268 (85.9)	44 (14.1)	< .001	
Comorbidities								
COPD	66 (9.9)	4 (2.0)	62 (13.3)	< .001	47 (8.4)	19 (17.6)	.007	
Hypertension	249 (37.3)	26 (12.9)	223 (48.0)	< .001	178 (31.8)	71 (65.7)	< .001	
Obesity	41 (6.1)	8 (4.0)	33 (7.1)	NS	30 (5.4)	11 (10.2)	NS	
Diabetes	72 (10.8)	8 (4.0)	64 (13.8)	< .001	48 (8.6)	24 (22.2)	< .001	
Chronic Kidney disease	52 (7.8)	1 (0.5)	51 (11.0)	< .001	25 (4.5)	27 (25.0)	< .001	
Ischemic Heart Disease	49 (7.3)	2 (1.0)	47 (10.1)	< .001	26 (4.7)	23 (21.3)	< .001	
Active cancer	39 (5.8)	5 (2.5)	34 (7.3)	.012	25 (4.5)	14 (13.0)	.002	
Immunodeficiency	8 (1.2)	0 (0.0)	8 (1.7)	NS	7 (1.3)	1 (0.9)	NS	
Symptoms onset > 6 days	244 (36.6)	74 (36.6)	170 (36.6)	NS	226 (40.4)	18 (16.7)	< .001	
Symptoms at ED admission								
Fever	545 (81.7)	154 (76.2)	391 (84.1)	.016	460 (82.3)	85 (78.7)	NS	
Dyspnea	249 (37.3)	60 (29.7)	189 (40.6)	.007	187 (33.5)	62 (57.4)	< .001	
Cough	371 (55.6)	130(64.4)	241 (51.8)	.003	336 (60.1)	35 (32.4)	< .001	
Conjunctivitis	10 (1.5)	8 (4.0)	2 (0.4)	.002	10 (1.8)	0 (0.0)	NS	
Pharingodynia	46 (6.9)	31 (15.3)	15 (3.2)	< .001	45 (8.1)	1 (0.9)	.006	
Headache	58 (8.7)	37 (18.3)	21 (4.5)	< .001	57 (10.2)	1 (0.9)	.001	
Fatigue	112 (16.8)	39 (19.3)	73 (15.7)	NS	102 (18.2)	10 (9.3)	.024	
Myalgia	77 (11.5)	46 (22.8)	31 (6.7)	< .001	74 (13.2)	3 (2.8)	.001	
Diarrhoea	96 (14.4)	41 (20.3)	55 (11.8)	.006	90 (16.1)	6 (5.6)	.004	
Anosmia	34 (5.1)	25 (12.4)	9 (1.9)	< .001	34 (6.1)	1 (0.9)	.003	
Ageusia/Disgeusia	58 (8.7)	37 (18.3)	21 (4.5)	< .001	58 (10.4)	0 (0.0)	< .001	
Syncope	10 (1.5)	2 (1.0)	8 (1.7)	NS	6 (1.1)	4 (3.7)	NS	
Vital Signs								
MBP, mmHg	92.4 ± 14.5	95.7 ± 12.6	91.6 ±14.8	.029	93.3 ± 13.3	89.4 ± 17.6	.044	
HR, beats per min	90.3 ±17.3	88.4 ±15.4	91.2 ± 18.1	NS	89.8 ± 16.6	93.1 ± 20.7	NS	
RR, breaths per min	19.8 ± 5.4	17.3 ±2.7	20.9 ±5.9	< .001	19.1 ± 4.6	24,2 ± 7.5	< .001	
SpO_2_, %	95.6 ± 4.4	97.9 ±1.5	94.6 ± 4.8	< .001	96.1 ± 3.8	92.2 ± 5.7	< .001	
BT, °C	37.2 ± 0.9	36.8 ±0.6	37.3 ± 0.9	< .001	37.1 ± 0.9	37.3 ± 0.9	.036	
Glasgow Coma Scale								
15	621 (93.1)	202 (32.5)	419 (67.5)	< .001	540 (87)	81 (13)	< .001	
≤14	46 (6.9)	0 (0.0)	46 (100)	< .001	19 (41.3)	27 (58.7)	< .001	
Kelly Scale								
grade 1-2	644 (96.6)	202 (31.4)	442 (68.6)	< .001	552 (85.7)	92 (14.3)	< .001	
grade 3-4	18 (2.7)	0 (0.0)	18 (100)	< .001	6 (33.3)	12 (66.6)	< .001	
grade 5-6	5 (0.7)	0 (0.0)	5 (100)	< .001	1 (20)	4 (80)	< .001	

On ABG we have found a standard pH value in all examined cohorts, while hypocapnia occurred in all patient categories with the exception of the discharged ones, who showed normal pCO_2_ values. As to the laboratory tests performed on admission, patients typically showed normal white blood cells (WBC), elevated C-reactive protein (CRP) (6.2 ± 7.4 mg/dL) and, lactate dehydrogenase (LDH) (289.6 ± 183.2 U/L). Lymphocytopenia was found in 40.3% of cases. Patients who required hospitalization or died, featured more significant laboratory abnormalities, including lymphocytopenia, neutrophils/lymphocytes ratio (N/L), D-dimer, LDH and, CRP, than those who got discharged. ABG and laboratory findings are listed in Table 2.

**Table 2:** Arterial blood gas and laboratory findings Data are mean ± SD, median (min-max) or n (%). PaCO_2_, PaO_2_: arterial carbon dioxide and oxygen tensions. P/F: arterial oxygen partial pressure/fractional inspired oxygen ratio. AaDO_2_: alveolar-to-arterial oxygen gradient. HCO_3_^-^: bicarbonate. FiO_2_: fractional inspired oxygen. WBC: white blood cells. N/L ratio: neutrophils/lymphocytes ratio. LDH: lactate dehydrogenase. CPK: creatine phosphokinase. CRP: C-reactive protein. PCT: procalcitonin. NS: not significant. ROX index: the ratio of oxygen saturation as measured by pulse oximetry/FiO_2_ to respiratory rate. FEU: fibrinogen equivalent units

	All patients (n=667)	Discharged (n=202)	Hospitalized (n=465)	p-value	Survivors (n=559)	Non-survivors (n=108)	p-value
Arterial Blood Gas Analysis							
pH	7.4 ± 0.6	7.4 ± 0.0	7.4 ± 0.7	< .001	7.4 ± 0.6	7.4 ± 0.1	NS
pO_2_, mmHg	75.9 ± 22.1	90.1 ± 13.6	69.7 ± 22.3	< .001	77.8 ± 19.9	65.9 ± 29.5	< .001
pCO_2_, mmHg	33.7 ± 6.7	35.5 ± 4.6	32.9 ± 7.3	< .001	33.6 ± 6.1	34.1 ± 9.3	NS
HCO_3_^- ^mmol/L	23.8 ± 3.4	24.1 ± 2.3	23.6 ± 3.8	.016	23.9 ± 2.8	23.1 ± 5.4	.007
Lactate, mmol/L	1.2 ± 1.1	1 ± 0.6	1.4 ± 1.3	< .001	1.1 ± 0.7	2.1 ± 2.2	< .001
FiO_^2^_	23.1 ± 9.9	21 ± 0.0	24.0 ± 11.8	< .001	21.8 ± 6.0	30.0 ± 19.4	< .001
P/F	347.0 ± 99.1	426.8 ± 63.5	312.4 ± 91.5	< .001	362.8 ± 90.5	261.7 ± 100.4	< .001
AaDO_2_	34.8 ± 17.7	18 ± 9.9	42.4 ± 14.9	< .001	32.8 ± 17.1	47.8 ± 15.6	< .001
AaDO_2_ expected for age	19.4 ± 4.7	15.6 ± 3.6	21 ± 4.2	< .001	18.5 ± 4.4	24.2 ± 2.8	< .001
Rox Index	24.3 ± 6.5	27.7 ± 4.2	22.8 ± 6.7	< .001	25.3 ± 5.9	18.6 ± 7.1	< .001
Laboratory tests							
WBC, 10^9^/L	7.9 ± 5.5	6.8 ± 2.9	8.3 ± 6.2	.028	7.4 ± 5.2	10.0 ± 6.5	< .001
Neutrophils, 10^9^/L	6.1 ± 6.4	4.4 ± 2.2	6.8 ± 7.3	< .001	5.7 ± 6.4	8.2 ± 6.0	< .001
Lymphocytes, 10^9^/L	1.6 ± 2.7	1.8 ± 1.1	1.6 ± 3.1	< .001	1.7 ± 2.9	1.3 ± 1.3	< .001
Eosinophils, 10^9^/L	0.1 ± 0.1	0.1 ± 0.2	0.0 ± 0.1	< .001	0.1 ± 0.1	0.0 ± 0.1	< .001
N/L ratio	5.8 ± 6.9	2.9 ± 1.9	6.9 ± 7.8	< .001	5.0 ± 5.9	9.8 ± 9.9	< .001
Platelets, 10^9^/L	220.4 ± 21.3	230.6 ± 71.5	216.4 ± 97.7	.001	221.2 ± 83.5	215.9 ± 123.5	.047
D-dimer, mg/L FEU	1.6 ± 4.0	0.4 ± 0.4	2.0 ± 4.6	< .001	1.1 ± 2.8	3.9 ± 7.3	< .001
Creatinine, mg/dL	1.1 ± 1.0	0.8 ± 0.6	1.2 ± 1.0	< .001	1.0 ± 0.8	1.6 ± 1.2	< .001
LDH, U/L	289.6 ± 183.2	206.1 ± 70.5	322.6 ± 202.5	< .001	262.4 ± 104.0	437.4 ± 363.5	< .001
CPK, U/L	83.0 (11-26758)	68.0 (30-276)	83.0 (11-26758)	NS	77.5(11-26758)	148.0 (16-953)	.012
Bilirubin, mg/dL	0.8 ± 1.9	0.6 ± 0.5	0.8 ± 2.0	< .001	0.6 ± 0.3	1.5 ± 4.2	< .001
CRP, mg/dL	6.2 ± 7.4	1.3 ± 2.3	8.1 ± 7.8	< .001	4.9 ± 6.0	13.1 ± 10.0	< .001
PCT, ng/mL	1.7 ± 11.3	0.1 ± 0.0	1.8 ± 11.6	.017	0.7 ± 3.5	5.8 ± 23.8	< .001
Lymphopenia,	269 (40.3)	35 (17.3)	234 (50.3)	< .001	203 (36.3)	66 (61.1)	< .001

Overall, AaDO_2_ values were significantly higher in the hospitalized versus discharged patients (21 ± 4.2 vs 15.6 ± 3.6; p< .001) and in non-survivor versus survivor patients (24.2 ± 2.8 vs 18.5 ± 4.4; p< .001).

Score creation and validation

Statistically significant variables at the univariate analysis were included in a multivariate regression. The results showed that male sex (OR, 1.856; IC 1.093-3.149 p <.022), age > 65 years (OR, 6.796; IC 3.622-12.753, p <.001), AaDO_2_ % (OR, 5.212; IC 3.373-8.054, p < .001), N/L (OR, 1.099; IC 1.039-1.235, p = .05) and increased levels of CRP (OR, 1.247; IC 1.130-1.376, p <.001) were associated with a higher rate of hospital admission (Table 3).

**Table 3:** Multivariate regression model for predicting hospitalization AaDO_2_: alveolar to arterial oxygen gradient. N/L: neutrophils/lymphocytes ratio. CRP: C-reactive protein.

Variables	Odds Ratio (95% CI)	p-value
Age > 65 years	6.796 (3.622 - 12.753)	< .001
Male sex	1.856 (1.093 - 3.149)	.022
AaDO_2_ percentage increase compared to expected for age	5.212 (3.373 - 8.054)	< .001
N/L	1.099 (1.039 - 1.235)	.05
CRP, mg/dL	1.247 (1.130 - 1.376)	< .001
Constant	1.115	

CovHos score was thus created using the formula:

$$(AaDO2 * 5.212) + (N/L * 1.099) + (CPR * 1.247)$$

adding 1.856 in case of male sex and 6.796 in case of age >65 years.

**Figure 1:**
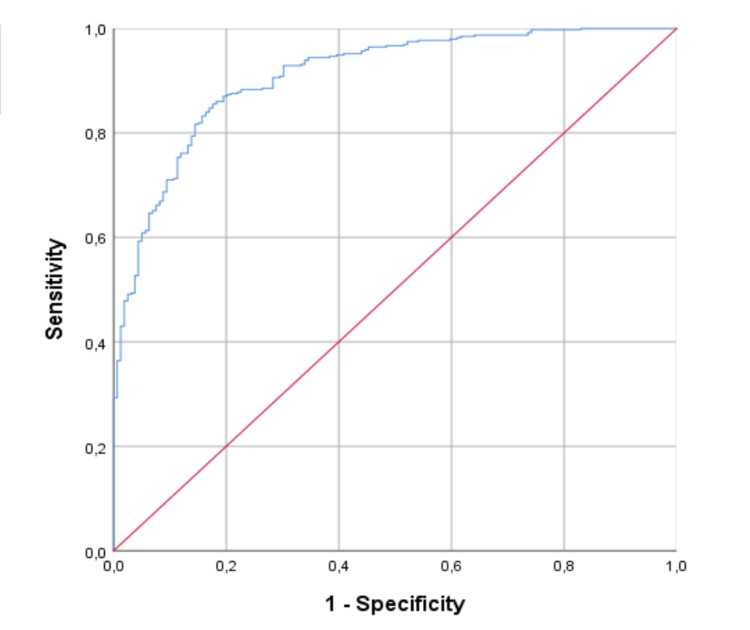
Receiver operating characteristic (ROC) analysis of CovHos Score in predicting hospitalization in COVID-19 patients

CovHos score, based on the multivariable regression, identified a cut-off of 12 points in predicting hospitalization with 85 % sensitivity and 82.4 % specificity (area under a receiver operating characteristic curve [AUROC] = 0.909, 95% CI 0.884 - 0.935, p < .001). Figure 1 shows ROC curve of CovHos score.

The ROC analysis allowed to define 22 points as the most accurate cut-off in predicting mortality: indeed, it showed a sensitivity of 79% and a specificity of 77% (AUROC 0.824; 95% CI 0.782-0.866).

**Figure 2:**
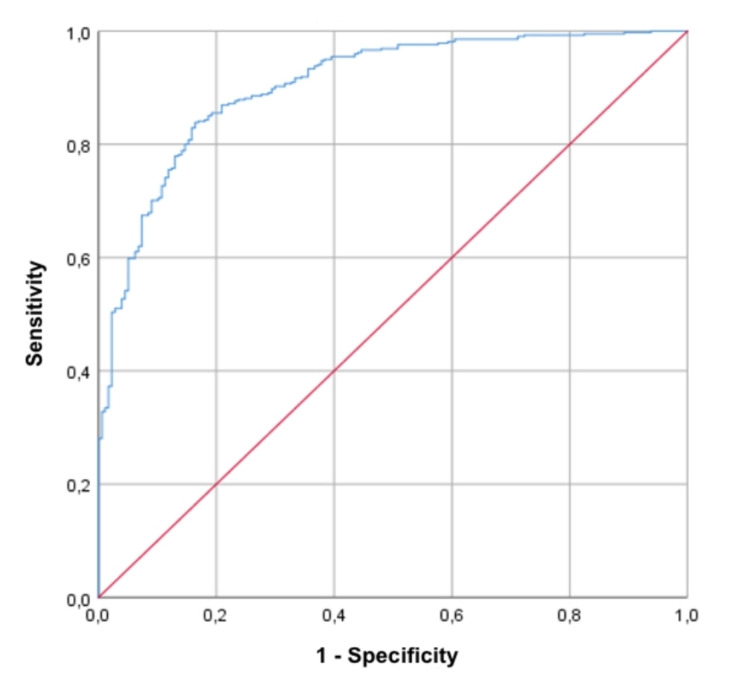
Receiver operating characteristic (ROC) analysis of CovHos Score in the validation group

We validated the score on 309 patients who fulfilled the same inclusion and exclusion criteria. Among these, 228 were hospitalized and 81 were discharged. The results showed that a CovHos score of 12 points has a sensitivity of 82% and a specificity of 74% in predicting the need for in-hospital treatment (p<0.001, AUROC 0.905, 95% CI 0.880-0.930). Figure 2 shows the ROC curve of the CovHos score in the validation group.

Moreover, the CovHos score of 22 points demonstrated a sensitivity of 71.4% and a specificity of 71.3% in mortality prediction (p<0.001, AUROC 0.820, 95%CI 0.778-0.863).

**Figure 3:**
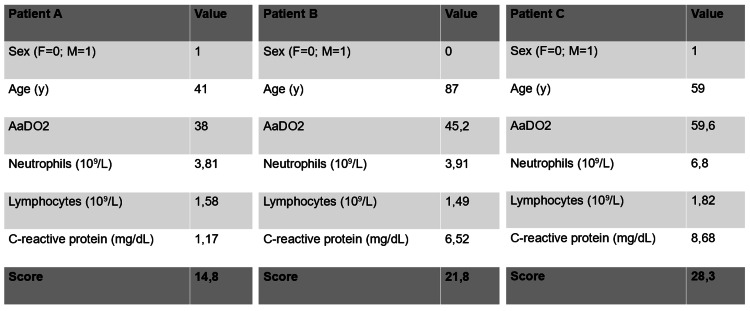
Examples of CovHos score application AaDO2 = Alveolar-to-arterial Oxygen Gradient; y = years

Examples of applicability of the CovHos score are presented in Figure 3.

Patient A was a 41-years-old man that came to ED with a fever for three days without dyspnea. The score was automatically calculated by filling values in a spreadsheet previously prepared. Despite good ABG values (pO_2_=75 mmHg, pCO_2_=29 mmHg), CovHos was 14.8 points. HRCT scan demonstrated the presence of spared ground-glass areas. The patient was admitted to the hospital and, during the hospital stay (six days) O_2_ was administered for the occurrence of dyspnea and worsening of ABG values. Patient B was an 87-years-old female patient admitted to ED for fever. Her CovHos score was 21.8 points. During her hospital stay (seven days) the patient remained eupnoeic and became afebrile. She was then discharged without complications. Patient C was a 59-years-old male deceased six days after intensive care admission. His CovHos score was 28.3 points.

The use of the CovHos score allowed to suggest the hospitalization of a young man who got worse in the following days. Moreover, by evaluating the score of patient B, indicative of hospitalization with a good prognosis, ED clinicians could foresee a relatively rapid discharge without complications. Lastly, the high CovHos score of patient C reflected the bad prognosis of a middle-aged man.

CovHos score in the study group

Out of 465 hospitalized patients, only one had a one-day observation for cardiological reasons, 72 patients (15.5%) had a length of hospital stay between two and five days, and 392 patients (84.3%) between six and 45 days. CovHos score was 18.6 ± 13 in the short hospital stay group and 24 ± 14 in the long hospital stay group (p < 0.05). Patients readmitted to hospital after a prior discharge (12 out of 202 discharged) showed a higher CovHos score (9.8 vs 3.8; p=0.006), higher AaDO_2_ (29 vs 16; p=0.001) and higher AaDO_2_ percentage increase (0.5 vs 0.03; p=0.007).

## Discussion

COVID-19 is a relatively unknown and challenging disease, featuring a broad spectrum of appearances and severity degrees. Based on our experience, and considering the sudden and sometimes unexpected evolution of this condition, it is crucial to early identify and stratify patients needing hospitalization or those who are more susceptible to adverse events, especially in a heterogeneous population setting like the ED. This is even more important in case of a pandemic, where hospital resources are limited. So far, scientific literature available on this subject has been mainly addressing severe cases involving acute respiratory failure until the development of ARDS. Although critical patients are more challenging from a clinical perspective, our data showed, like those from the general literature [11,12] that at the time of ED admission the majority of COVID-19 cases was mild. Therefore, the aim of this study is to identify risk factors for hospitalization in adults affected by COVID-19 presenting at the ED, in order to build a score as a quick and effective aid to the physician in determining the need for hospitalization, and potentially, the correct intensity of care. A similar approach was used by Bartoletti et al. [13] who demonstrated that age, obesity, body temperature, RR, lymphocytes, CRP, creatinine, and LDH>350 IU/L, considered together in the so-called PREDI-CO score, have good accuracy in predicting severe respiratory failure (SRF) (AUROC 0.89, sensitivity 80% and specificity 76% at a score>3 points). However, the only parameter influenced by respiratory dynamics in such a score is RR, without any attention to ABG parameters. We have already demonstrated the utility of the ROX index, which is defined as the ratio of peripheral oxygen saturation and the fraction of inspired oxygen to respiratory rate, in predicting hospitalization and mortality in patients with a diagnosis of COVID-19 in the ED [14]. However, even such an approach does not take into consideration ABG parameters.

The analysis of ABG findings showed that, although a large number of patients had hypocapnia, pH values were regular. Hypoxemia was more frequent among severe patients and since their first medical contact, they generally underwent oxygen therapy. Considering the well-known importance of ABG in determining the level of respiratory impairment, we analyzed P/F values, which were significantly lower in hospitalized patients and non-survivors. However, in order to have a more accurate identification of an incipient respiratory failure, we considered AaDO_2_, which seemed to play a relevant clinical role. It is well known that due to the sigmoid shape of the oxygen dissociation curve, the accuracy of SpO_2_ readings above 90% becomes uncertain. Given the flatness of the upper oxygen-dissociation curve, a pulse oximetry reading of 95% may denote a PaO_2_ anywhere between 60 and 200 mmHg. In this connection, AaDO_2_ allows for a more precise evaluation of the pathophysiological basis of hypoxemia than the more widely used PaO_2_/FiO_2_ [15], giving support to the differential diagnosis with other respiratory diseases.

Multivariate regression showed that age>65, male sex, AaDO_2_%, N/L, and CRP values were the main statistically significant variables able to identify COVID-19 patients needing an in-hospital observation period.

In order to help emergency physicians who first evaluate patients suspected of COVID-19 at the time of ED admission, we built a score based on the five above-mentioned variables. CovHos score could be a useful tool to identify patients needing admittance to a hospital ward. In our context, a CovHos score higher than 12 points had 85 % sensitivity and 82.4 % specificity in the identification of hospital admission. A different cut-off may be considered in other health care systems, depending on local resources of a pandemic that devastated global healthcare systems. Indeed, emergency physicians should decide every day to hospitalize or to discharge patients on a probabilistic basis in many clinical situations, considering together demographic, clinical, laboratory, and radiological variables. In some contexts, precautionary hospitalization may be unrealistic due to limited offers or disproportionate demand. For these reasons, we suggest a cut-off that appears a good compromise between patient safety and resource sparing; however, the score increase in severe conditions does not impede small variations of proposed cut-off values in different situations. Moreover, the CovHos score may be used to allocate patients in the appropriate setting of care even in wards other than intensive care units. Worth to remark that the higher CovHos score is associated with a longer hospital stay and with mortality.

Some limitations should be noted, such as the monocentric nature of our study. We are also aware that we have not defined beforehand the reference standard to determine the hospitalization need, which was decided by the association of clinical judgment, patients’ characteristics, and medical reports. However, the low number of patients re-admitted to the hospital after a prior discharge and the extremely low prevalence of one-day hospital stay corroborate our appropriateness of hospitalization evaluation.

## Conclusions

The CovHos score could be a useful tool to help physicians select patients who might be suitable for home management, especially in a shortage of hospital beds. Although we believe medical judgment still remains crucial in the final clinical decision, our aim was to create a useful score for ED management of COVID-19 patients. A high CovHos score should increase clinicians' attention in discharging patients. The relation between the CovHos score and patient worsening is also evident with respect to mortality and length of hospital stay. Further studies especially on a larger scale from different places would help in understanding and appreciating the validity of the score even better. 
